# A Case of Tumor

**Published:** 1883-04

**Authors:** 


A Case of Tumor of the skull and bladder was reported, and the specimens presented, by Mr. Clutton, at a meeting of the Pathological Society of London (*London Lancet*). The patient was first seen in 1877, and had at that time a swelling over and fixed to the left parietal bone, which was soft in the center and had an indurated margin. This was opened and a large opening was found in the skull. He died about six months afterwards from the profuse discharge and hectic. He suffered from severe neuralgia, but there was no paralysis nor any symptoms of trouble in the bladder. The post-mortem disclosed an opening in the skull three inches across, the ulcer in the scalp being four by five inches. The exposed dura mater was thick and rough, and in its center was an opening about the size of a florin, beneath which the brain showed commencing hernia. A tumor as large as an orange occupied the left side of the body of the bladder, both inside and outside, its margin being well defined. Microscopical examination of the tumor of the head showed an alveolar structure, with large cells having distinct nuclei and nucleoli, with interalveolar small round and spiral cells. It was considered to be an alveolar sarcoma. The bladder tumor was composed of round and oval cells like those of sarcoma. As to which was the primary growth in this case, or whether they were entirely independent of each other, were questions about which there was a difference of opinion.—*Weekly Med. Review.*

